# Combination of CNTs with Classical Drugs for Treatment in Human Colorectal Adenocarcinoma (HT-29) Cell Line

**DOI:** 10.3390/nano13131933

**Published:** 2023-06-25

**Authors:** Sara Abreu, Nuno Vale, Olívia Salomé G. P. Soares

**Affiliations:** 1 Laboratory of Separation and Reaction Engineering-Laboratory of Catalysis and Materials (LSRE-LCM), Department of Chemical Engineering, Faculty of Engineering, University of Porto, Rua Dr. Roberto Frias, 4200-465 Porto, Portugal; 2 ALiCE—Associate Laboratory in Chemical Engineering, Faculty of Engineering, University of Porto, Rua Dr. Roberto Frias, 4200-465 Porto, Portugal; 3 OncoPharma Research Group, Center for Health Technology and Services Research (CINTESIS), Rua Dr. Plácido da Costa, 4200-450 Porto, Portugal; 4 CINTESIS@RISE, Faculty of Medicine, University of Porto, Alameda Professor Hernâni Monteiro, 4200-319 Porto, Portugal; 5 Department of Community Medicine, Health Information and Decision (MEDCIDS), Faculty of Medicine, University of Porto, Rua Dr. Plácido da Costa, 4200-450 Porto, Portugal

**Keywords:** carbon nanotubes, tacrine, ethionamide, 5-fluorouracil, HT-29, drug repurposing, colon cancer

## Abstract

Due to the increase in new types of cancer cells and resistance to drugs, conventional cancer treatments are sometimes insufficient. Therefore, an alternative is to apply nanotechnology to biomedical areas, minimizing side effects and drug resistance and improving treatment efficacy. This work aims to find a promising cancer treatment in the human colorectal adenocarcinoma cell line (HT-29) to minimize the viability of cells (IC_50_) by using carbon nanotubes (CNTs) combined with different drugs (5-fluorouracil (5-FU) and two repurposing drugs—tacrine (TAC) and ethionamide (ETA). Several CNT samples with different functional groups (-O, -N, -S) and textural properties were prepared and characterized by elemental and thermogravimetry analysis, size distribution, and textural and temperature programmed desorption. The samples that interacted most with the drugs and contributed to improving HT-29 cell treatment were samples doped with nitrogen and sulfur groups (CNT-BM-N and CNT-H_2_SO_4_-BM) with IC_50_ 1.98 and 2.50 µmol∙dm^−3^ from 5-FU and 15.32 and 15.81 µmol∙dm^−3^ from TAC. On the other hand, ETA had no activity, even combined with the CNTs. These results allow us to conclude that the activity was improved for both 5-FU and TAC when combined with CNTs.

## 1. Introduction

Over the years, there has been an increase in the number of patients with new types of cell cancer [1]. In 2020, almost 10 million people died worldwide, including 1.80 million deaths from lung cancer and 916 thousand deaths from colorectal cancer. In addition, new cancer cases appeared, with breast cancer in first place, lung cancer in second, and colorectal cancer in third, with 1.93 million [2].

During growth, humans develop certain “normal” cells that undergo structural, morphological, and functional modifications, which can induce the proliferation of malignant cells with cell cycles. This cellular modification is called extracellular modification transition (EMT), i.e., it describes a cellular transition of the epithelial tissue—one of the four types of human tissues—at which point the cells gradually lose their epithelial characteristics, leading to the onset of cancer. These cellular changes may be related to the environment, such as the pollution associated with pathogenic infections and exposure to ultraviolet (UV) radiation, as well as unhealthy habits, such as smoking, food deficit, daily stress, and lack of physical activity [1,3,4,5,6,7].

With technological advances, treatments to combat tumor cells were discovered, highlighting chemotherapy, radiotherapy, surgery, targeted therapy, and immunotherapy. However, these therapies have some limitations, such as high cytotoxicity for “normal” cells, low specificity, possible drug resistance, short half-life, low solubility, and potential drug resistance [8,9,10]. Despite these limitations, it has been possible to study other alternative methods, such as combination treatments and conjugation with nanomedicine, which are based on the use of nanomaterials (with a size between 1 and 1000 nm) applied in health care, in different functions such as molecular imaging, biomarker mapping, direction and diagnosis, drug delivery, target therapy, and gene delivery [4,6,8,10,11,12,13], whereas some of the most used nanomaterials are polymeric nanoparticles, liposomes, solid lipid nanoparticles (SLN), metallic nanoparticles, magnetic nanoparticles, and carbon nanotubes (CNTs) [4,8,10,12,13]. 

CNT can result in two different structures depending on their growth conditions during the synthesizing process, such as single (SWNT) or multiwalled (MWNT) graphene, and can be distinguished by their high specific surface area; greater mechanical strength, highlighting SWNTs, which provide higher thermal conductivity; and chemical stability compared to other nanoparticles usually used as nanocarriers [8,9,12,13,14]. However, their use brings some challenges, such as low solubility, the release of possible toxic ions during synthesis [15], the strong hydrophobicity of CNTs that leads to aggregation and precipitation in aqueous solutions, and increased viscosity. Two possible strategies to surpass challenges are using pure CNTs, which avoids the release of toxic metals in a biological environment, and functionalizing the CNTs, which improves their solubility and stability [8,10,12,13,14,15]. A study by Sayes et al. [16] revealed that chemically functionalized CNTs were less cytotoxic than non-functionalized ones. Therefore, functionalization and textural modification of CNTs favor their dispersion, can decrease their size, and, by modifying their surfaces, can induce biocompatible groups, reducing their toxicity [13,14,15]. In contrast, very small sizes can also lead to toxicity due to their high surface area, i.e., although the contact area with cell membranes is high, the probability of adsorbing toxins also increases [8,10,14,17]. When the drugs are adsorbed onto CNTs and administered into the system, they must have high cell specificity, good biocompatibility, and adequate size so that they can overcome biological and physiological (cell membranes), or mechanical and physicochemical (enzymes) barriers [6]. CNTs generally enter cells via an endocytosis mechanism (pinocytosis or phagocytosis) or by passive diffusion, as with functionalized CNTs [18,19]. The selection of the mechanism of cellular action is affected by several factors depending on the physicochemical properties of CNTs (size and shape; surface charge; and hydrophobicity) and cell type, highlighting the surface of nanoparticles, which must be hydrophobic to increase the affinity and favor their adsorption in cells [18,19]. According to an article by Elidamar Lima et al. [19], the biocompatibility of CNTs can be improved when functionalized with acids.

Drug repurposing is a recent strategy increasingly used in cancer therapy research [20] involving drugs that the Food and Drug Administration (FDA) has already approved, i.e., disused drugs or new ones that may have better activity when combined with nanomaterials. Since these drugs were already approved, the process is faster and less costly than when using new drugs, making it more advantageous [5,21,22]. 

**Figure 1:**
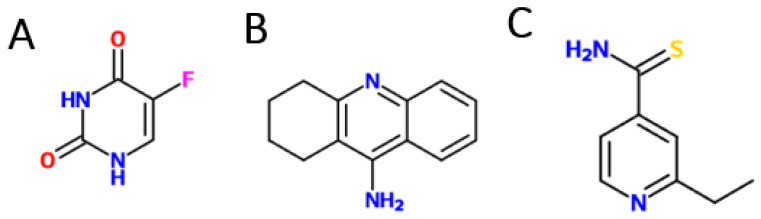
Molecular structures of drugs: (**A**) 5-Fluorouracil. (**B**) Tacrine. (**C**) Ethionamide [23].

In this study, CNTs with different modifications, chemical and textural, were combined with individual drugs, 5-fluorouracil (5-FU), a reference drug already used in the treatment of HT-29 cells, Figure 1A, and two repurposed drugs (tacrine (TAC) used to treat Alzheimer’s disease, Figure 1B, and ethionamide (ETA) used in the treatment of tuberculosis, Figure 1C) and applied to the human colorectal adenocarcinoma cell line (HT-29). This combination consisted of a dispersant solution of CNTs, to which the drug was added at a 1:1 ratio for 48 h via the adsorption mechanism. These nanomaterials have been overtaken by others due to the above-mentioned characteristics, and many researchers have worked on metallic and organic nanoparticles, with these materials being the most used in the treatment of tumor cells, setting aside CNTs. Despite this, the general idea is to improve their specificity and encapsulation of drugs by functionalizing them, focusing on the innovative part of the work, which is the repurposing of drugs in the treatment of HT-29 cells.

To date, studies in which drugs are combined with CNTs for treating HT-29 cells are scarce. Some studies have reported graphene oxide (GO) combined with 5-FU for cancer treatments. In a study by Zhang et al. [24], 5-FU was encapsulated in GO functionalized with sodium alginate and tested in HT-29 cells. The results were satisfactory, with loading capacity and efficiency of 24.40% and 32.53%, respectively, and the activity was identical in both situations; however, 5-FU/GO was able to inhibit metastasis effectively and significantly suppress tumor growth. The repurposing drugs have not yet been connected to CNTs and tested in HT-29 colon cells; however, both were associated with other nanoparticles. For example, TAC is commonly used to treat brain disease, and it was tested with CNTs, lipid-based nanoparticles, etc. [25,26,27,28], and ETA is related to the treatment of lung disease, and it was tested with polymeric nanoparticles, lipid-based nanoparticles, etc. [29,30,31]. Due to the lack of results involving CNTs connected to these drugs in treating HT-29 cancer cells, research in this direction can be a great innovation and make a difference in the future.

## 2. Materials and Methods

### 2.1. Sample Preparation

Commercial CNTs (MWCNTs, Nanocyl—NC3000) with a purity of 95% (CNT sample) was used as a starting material. According to the supplier, these nanotubes have an average diameter of 9.5 nm and an average length of 1.5 µm. A sample with different textural properties was prepared using a ball milling approach by milling the CNT sample for 4 h at 15 s^−1^ (CNT-BM sample) [32]. 

A N-doped sample was prepared by mixing a mass of commercial CNTs ≈ 0.6 g with ≈0.39 g of C_3_H_6_N_6_ (ALDRICH Chemistry, 99%, St. Louis, MO, United States), which functions as a nitrogen precursor, in a ball mill (Retsch MM 200) for 4 h at 15 s^−1^. Next, the sample was subjected to heat treatment in an oven at 600 °C for 1 h with a ramp of 10 °C∙min^−1^ and a N_2_ flow rate of 100 cm^−3^∙min^−1^ (CNT-BM-N sample) [32]. 

O or S-containing samples were obtained by oxidation in the liquid phase of the commercial CNTs with a 7 mol∙dm^−3^ solution of HNO_3_ (Supelco, 65%) with a volume of 300 cm^−3^ or with H_2_SO_4_ solution (VWR BDH CHEMICALS, 95%, Radnor, PA, USA) with a volume of 150 cm^−3^. The solution with HNO_3_ was boiled at reflux, heated on a heating mantle for 3 h, and then cooled to room temperature, whereas the solution with H_2_SO_4_ was subjected to reflux, heated at a temperature of 50 °C for 4 h, and subsequently cooled to room temperature. Both were then washed with distilled water until reaching a neutral pH and finally dried in an oven at 100 °C for at least 24 h (CNT-HNO_3_ and CNT-H_2_SO_4_ samples). The sample with HNO_3_ was subjected to heat treatment to remove some functional O-containing groups in an oven at 600 °C for 1 h with a ramp of 10 °C∙min^−1^ and a N_2_ flow rate of 100 cm^3^∙min^−1^ (CNT-HNO_3_-600 sample) [32]. 

All samples were mechanically treated and milled for 4 h at 15 s^−1^ (CNT-HNO_3_-600-BM, CNT-HNO_3_-BM, and CNT-H_2_SO_4_-BM samples) [32].

### 2.2. CNT Characterization Techniques

The material’s size was characterized using laser scanning confocal microscopy (LSCM) on a Coulter LS230 (Beckman Coulter, Inc., Brea, CA, USA). LSCM consists of a beam of light passing through the pinhole, where there is an adjustment in the wavelength to excite the electrons/molecules in the sample. Then, the visualization is captured by the scanner, and the length of the nanoparticles is determined.

The textural characterization was based on N_2_ adsorption isotherms at −196 °C performed on a Quantachrome NOVA 4200e (Boynton Beach, FL, USA). The samples were degassed at 120 °C for 3 h. In this way, it was possible to determine parameters such as the specific surface area (*S_BET_*) and the total pore volume (*V_pT_*). The distribution of pore size was calculated using non-local density functional theory (NLDFT) [32]. For each of the parameters, different models were applied. In the determination of *S_BET_*, the Brunauer, Emmett, and Teller (BET) model was used, which describes the physical adsorption in type II isotherms at relative pressures between 0.05 and 0.3. The *V****_pT_*** was determined through the adsorption of N_2_ at *P/P*0 = 0.95 [32].

Thermogravimetric analysis (TGA) is a qualitative analysis that evaluates the thermal stability of the sample passing through three phases: dehydration, burning of Volatile compounds (VCs), and burning of fixed carbon. It was performed by heating the samples from 50 to 900 °C, with a heating ramp of 10 °C∙min^−1^; passing, at the final temperature, a nitrogen current for 7 min; and then an air current for 13 min, using STA 409 PC/4/H Luxx NETZCH equipment (Selb, Germany) [32].

Elemental analysis (EA) was carried out to quantify carbon, hydrogen, nitrogen, and sulfur by combustion of the materials at 1050 °C, using Elemental GmbH MICRO equipment in CHNS mode, whereas oxygen analysis was determined by pyrolysis of the materials at 1450 °C utilizing OXY equipment from Elemental GmbH (Kalkar, Germany) [32]. 

The oxygen-containing groups were characterized by temperature programmed desorption (TPD) with AMI 300 characterization apparatus (Altamira Instruments, Pittsburgh, PA, USA) connected to a Dymaxion Dycor mass spectrophotometer (Ametek, Pittsburgh, PA, USA). The samples, weighing 0.1 g, were placed in a U-shaped quartz tube located inside an electric furnace and heated to 1100 °C at 5 °C∙min^−1^ with a constant helium flow rate of 25 cm^3^∙min^−1^. The amounts of CO and CO_2_ released during thermal analysis were monitored, and these gases were calibrated at the end of each analysis [32].

### 2.3. Drugs

The drugs studied were 5-FU, TAC, and ETA. 5-FU (C_4_H_3_FN_2_O_2_, Sigma-Aldrich, ≥99%, St. Louis, MO, USA), with a molecular weight of 130.08 g∙mol^−1^, is soluble in methanol and water and insoluble in benzene and chloroform, is a stable compound, and has a pKa of 8.02 [33]. TAC (C_13_H_14_N_2_-HCl-xH_2_O, Sigma-Aldrich, ≥99%), with a molar mass of 234.72 g∙mol^−1^, is soluble in methanol and has a pKa of 9.8 [34]. ETA (C_8_H_10_N_2_S, Sigma Aldrich) is a thioamide-derived antibacterial with a molecular molar weight of 166.24 g∙mol^−1^; is soluble in methanol, ethanol, and propylene glycol; and has a pKa of 4.49 [35].

### 2.4. Study of the Adsorption of Drugs in CNT

To better understand the adsorption of each drug in the different samples, a kinetic assay was performed for 48 h. The methodology consisted of weighing 2.0 ± 0.1 g of each CNT sample, in Eppendorf tubes and putting them in contact with a solution of each drug, individually, at 250 mg∙dm^−3^ with methanol (CH_3O_H, VWR CHEMICALS, ≥99.9%) for [0:00; 0:15; 0:30; 1:00; 2:00; 4:00; 18:00; 24:00; 47:00] h. The Eppendorf tubes were put on a P SELECTA ROTABIT shaker at 130 rpm. Each Eppendorf tube was removed and placed in a VWR MicroStar12 centrifuge at 30 rpm for 15 min. Finally, ≈0.8 cm^−3^ of the supernatant was removed and placed in vials, which were then read using an analytical method, high-performance liquid chromatography (HPLC). HPLC methods were optimized for each drug based on the literature [36,37,38] and performed in equipment (Hitachi Elite LaChrom HPLC, Tokyo, Japan) equipped with a UV detector and a C_18_ column (YMC Hydrosphere, 250 mm × 4.6 mm). Thus, the method selected involved a ratio of 20% sodium phosphate buffer solution monobasic monohydrate at 0.05 M (H_2_NaO_4_P-H_2_O, SIGMA-ALDRICH, ≥98%) and 80% acetonitrile (CH_3_CN, VWR CHEMICALS, ≥99.95%); an injection volume of 10 µL in TAC and 20 µL in ETA and 5-FU; at 1 mL∙min^−1^; and wavelengths for TAC, ETA, and 5-FU of 243, 254, and 266 nm, respectively.

### 2.5. Cell Lines and Cultures

The HT-29 cells (ATCC HTB-38, American Type Culture Collection, Manassas, VA, USA) were grown in McCoy’s 5A Modified Medium with 10% fetal bovine serum (FBS) at 37 °C in a humidified atmosphere with 95% air and 5% CO_2_ [39]. Cell growth took place in 25 cm^2^ t-flasks with adherent polymer together with the respective medium. The cells were maintained every 3 days, and trypsinization was performed after reaching cell concentrations between 70% and 80% [5]. Initially, the medium was removed, and 4 mL of phosphate-buffered saline (PBS) was used to remove the remaining medium. Next, 500 µL of 0.25% trypsin-EDTA was added and left in the oven at 37 °C for 5–8 min to disaggregate the cells from the polymer. After disconnecting from the polymer, 3 mL of the respective medium was added to inactivate the trypsin enzyme. In a new t-flask, 5 mL of the new medium was placed, and 100 µL cells were added [5].

### 2.6. Cell Treatment

The drugs were dissolved in the solvent dimethyl sulfoxide (DMSO) (Merck, Rahway, NJ, United States) with concentrations that ranged from 0.1 to 100 mM. The DMSO was diluted 1000× in culture medium so that the DMSO was at a concentration of 0.1% in the cells and was not toxic to them [5]. The maximum drug concentration tested in the cells was 100 µM.

### 2.7. CNT Dispersion

The CNT dispersion methodology was applied to the samples alone and in combinations with the drugs. This required the addition of a dispersing agent to ensure the dispersion of the nanoparticles and prevent their aggregation. Various dispersants can be used in CNTs, such as Pluronic F-127 [40,41,42,43,44]. F-127 is characterized as a non-ionic polyether surfactant or copolymer and is widely used to disperse hydrophobic nanoparticles in suspension [45]. A solution of F-127 of 160 μg∙cm^−3^ was prepared with distilled water. Individual CNT samples were prepared with F-127 at concentrations of [2.5; 5; 10; 20; 40; 80] μg∙cm^−3^, sonicated for 10 min, and stirred in the solution [45]. After selecting the best sample concentration, each sample was combined with each drug at different concentrations of [1; 10; 50; 100] mM, at a 1:1 volume ratio, obtaining final drug concentrations of [0.5; 5; 25; 50] mM. Then, the solution was sonicated for 10 min, stirred, and diluted in 500× culture medium. The dispersion of the individual and combined samples should be prepared on the day of cell treatment of the MTT (3-4,5-dimethylthiazol-2-yl-2,5-diphenyltetrazolium bromide) assay because, even with the action of the surfactant and ultrasound, the CNTs are not dispersed for long periods [42].

### 2.8. MTT Assay

After maintaining the cells and before placing them in new t-flasks, on day 1 the cells were plated in 96-well plates. For this, a cell count was carried out in which trypan blue dye was added to the cells at a 5:1 ratio. The count was performed for ×5 and ×1000 culture media. Next, ≈10,000 cells per well were plated. Finally, the cells were incubated for 24 h at 37 °C in a humid atmosphere with 95% air and 5% CO_2_. On day 2, the medium was aspirated, and the cells were treated with a solution of drug or drug combined with CNTs by adding 200 µL in each well. After 48 h, on day 3, the medium in each well was removed, and 100 µL of MTT was added. The plates were protected from light and placed at 37 °C for 3 h. After 3 h, 100 µL of DMSO was added to dissolve the crystals formed, and the absorbance was then read in a plate reader at 570 nm [5].

### 2.9. Cell Morphology Visualization

Cells were observed and photographed after each treatment, using Leica DMI6000 B Automated Microscope (Leica, Wetzlar, Germany) and using a Leica LAS X software (v3.7.4) [5].

### 2.10. Data Analysis

The results of the textural characterization were analyzed in NovaWin software (version 11.02, Quantachrome Corporation, Boynton Beac, FL, USA), and those of the TPD were analyzed in OriginPro 8.6 32Bit software, in which the determination of the area of the peaks was used to quantify the amounts of CO and CO_2_. The experimental cell data were analyzed in GraphPad Prism 8 software (GraphPad Inc. software, San Diego, CA, USA) with one-way analysis of variance (ANOVA). To analyze the evolution of cell viability along the concentrations, a method was developed where the intervals of the mean had 95% confidence intervals, and the differences were considered significant at *p* < 0.05. After this, the viabilities of treated cells were normalized with the viability of control cells, and afterwards cell viability fractions were plotted as a function of drug concentration on a logarithmic scale, where least-squares regression was applied to determine IC_50_.

## 3. Results and Discussion

### 3.1. Characterization Techniques

**Figure 2:**
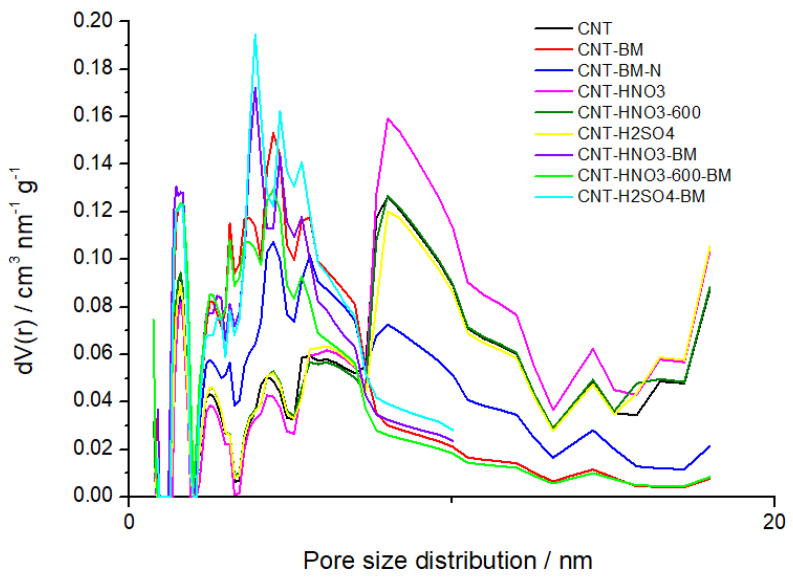
Pore size distribution obtained by NLDFT of the CNT samples.

The textural properties of each sample were analyzed by determination of particle size (*d*) and nitrogen adsorption–desorption isotherms at −196 °C to determine parameters such as the specific surface area (*S_BET_*) and the total pore volume (*V_pT_*) in Table 1 and pore size distribution as presented in Figure 2.

Concerning the sample size, *d* (µm), it is important to emphasize that these values correspond not to the dimensions of individual CNTs but to the agglomerates. Thus, comparing the CNT and unmilled samples (CNT-HNO_3_; CNT-HNO_3_-600; CNT-H_2_SO_4_) with the milled samples (CNT-HNO_3_-BM; CNT-HNO_3_-600-BM; CNT-H_2_SO_4_-BM) exhibits a decrease in size to half and quarter, due to the milling process to which they were subjected, leading to higher *S_BET_* and a smaller pore volume. In the case of the oxygenated samples (CNT-HNO_3_; CNT-HNO_3_-600), in the first sample, a reduction in size and an increase in surface area characteristic of purification of the nanotubes occurs, while in the second sample, as functional groups were removed due to the heat treatment, compared to CNT-HNO_3_, there was an increase in their size and consequently a decrease in area since the surface functional groups could promote some agglomeration of the tubes and block the access of nitrogen to inner cavities. Finally, in the CNT-BM-N sample, the addition of nitrogen groups caused a decrease in its area and thus could block nitrogen access to the pores [32,46]. As expected, the micropore volume was 0 cm^3^∙g^−1^ in all samples, since CNTs are classified as mesoporous materials with pore size < 50 nm and >2 nm and characteristically exhibit type II isotherms [47]. Finally, Figure 2 shows a higher intensity of pore size below 10 nm in milled samples (CNT-BM; CNT-HNO_3_-BM; CNT-HNO_3_-600-BM; CNT-H_2_SO_4_-BM) and a higher intensity of pore size near 10 nm in unmilled samples (CNT-HNO_3_; CNT-HNO_3_-600; CNT-H_2_SO_4_), which is in line with abovementioned findings, highlighting the reduction in size after milling [48]. Moreover, the reduction in their size was revealed to be an important parameter in the delivery and treatment of tumor cells [32].

EA, TGA, and TPD characterization techniques were used to analyze the chemically functionalized samples. The only difference between milled samples (CNT-HNO_3_-BM; CNT-HNO_3_-600-BM; CNT-H_2_SO_4_-BM) and unmilled (CNT-HNO_3_; CNT-HNO_3_-600; CNT-H_2_SO_4_) samples is the mechanical treatment that they underwent, so this aspect was not analyzed. To quantify the chemical elements nitrogen (N), sulfur (S), and oxygen (O) in each sample, EA was used. According to Table 2, the CNT-BM-N sample showed the highest value of N at 4.3%. A study by Soares et al. [49] determined the amounts of each element and obtained 3.5% N, like this work. The CNT-H_2_SO_4_ sample contains 0.2% S and 1.4% O, corresponding to sulfonic groups. As for the oxygenated samples, CNT-HNO_3_ and CNT-HNO_3_-600, they contained 1.2 and 0.5% O. The TGA evaluates the thermal stability of the samples. In Table 2, the CNT-BM-N sample presents the highest value of volatile compounds (VCs) due to the introduction of N-groups. Samples CNT-HNO_3_, CNT-HNO_3_-600, and CNT-H_2_SO_4_) also present a high quantity of volatile compounds due to the presence of oxygenated groups in these samples. TPD analysis allows the amounts of CO and CO_2_ released from the samples to be quantified, which is related to the oxygenated groups each sample may contain, according to the temperature at which these groups are released. In Table 2, the values of the amounts of CO and CO_2_ in the CNT sample were 334 and 176 µmol∙g^−1^, while in the CNT-HNO_3_ and CNT-HNO_3_-600 samples they were 1002 and 440 µmol∙g^−1^ and 900 and 360 µmol∙g^−1^, respectively, revealing the presence of oxygenated groups in the different samples such as carboxyl, lactone, phenol, carbonyl, anhydride, ether, quinone, and pyrone [50]. Because of the heat treatment at 600 °C, on the sample CNT-HNO3-600, some of the groups were eliminated, such as carboxyl, lactone, anhydride, and some of the phenol group, which caused a lower number of oxygenated groups compared to the CNT-HNO_3_ sample. Hence, the amounts of CO and CO_2_ in the CNT-HNO_3_-600 sample were lower than in the CNT-HNO_3_ sample [50]. 

### 3.2. Assay for Drug Adsorption on CNTs

The aim of the adsorption assay was to study the adsorption kinetics of each drug in the different samples over 48 h. This study was qualitative and was intended to evaluate the behavior of each sample with each drug before starting the biological tests. 

The functionalized samples can be connected to drugs by chemical interaction. **5-FU** is an aromatic compound containing C=O, N-H, and C-H bonds that bind to -COOH and -OH groups of the functionalized CNTs (CNT-HNO_3_; CNT-HNO_3_-600; CNT-H_2_SO_4_), forming hydrogen bonds with stronger interactions than hydrophobic ones [51]. The same occurs with samples containing N-groups (CNT-BM-N), whose interactions lead to the formation of -CN groups. According to a study by Kamble et al. [52], the encapsulation efficiency of 5-FU in CNTs with functional groups is higher than with CNTs without functionalization. **TAC** consists of three hexagonal cycles with an amine (NH_2_) and many C=C bonds, so there is a more significant interaction with CNTs via hydrophobic bonds [25]. While 5-FU can connect mostly by hydrogen bonds, hydrophobic bonds are weaker. As a result, TAC had more difficulty connecting to the samples than 5-FU. ETA has an amine group and sulfur, such that it behaves identically to TAC. However, since TAC has three aromatic rings and ETA only one, the former can interact more than the latter. 

The milled samples without functionalization had more contact with the drugs in the ball mill because of mechanical treatments, where the nanotubes are broken at their ends, managing to adsorb the drugs onto the graphene sheet layers due to their hydrophobic interactions [52,53,54].

Since, in the second situation, the milled samples adsorbed the drugs better, it was decided to mill functionalized samples to favor their interaction (CNT-HNO_3_-BM, CNT-HNO_3_-600-BM, and CNT-H_2_SO_4_-BM). These samples were then tested in the cells. Another advantage of using milled samples in biological assays is that they are smaller in size, facilitating their penetration into the cells [8,18,19,55,56].

### 3.3. Cell Assays

At first, the activity of the different free drugs in HT-29 cells was evaluated, as detailed in Section 3.3.1. Then, the toxicity of the samples was studied, as outlined in Section 3.3.2, and finally, each sample was combined for a given concentration, with each drug at different concentrations, as shown in Section 3.3.3.

#### 3.3.1. Cell Treatment of Free Drugs

Each drug was studied at different concentrations over 48 h to determine the IC_50_.

**Figure 3:**
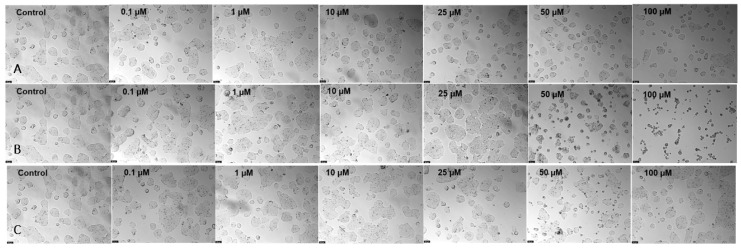
Microscopic cellular visualization of HT-29 cells after 48 h of incubation with drugs. (**A**) Incubation with 5-fluorouracil. (**B**) Incubation with tacrine. (**C**) Incubation with ethionamide at different concentrations of [0.1–100] µM.

**Figure 4:**
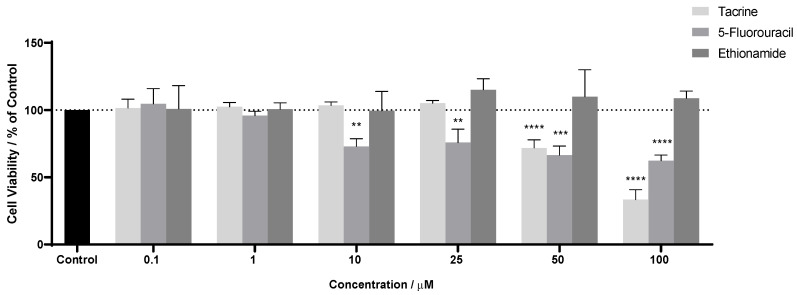
The effects of drugs on HT-29 cells. Cells were cultured in the presence of increasing concentrations of each drug, and after 48 h, the MTT assay was performed to measure the cellular viability. Values are expressed in percentage of control and represent means ± SEM. Each experiment was conducted three times independently (*n* = 3); ** statistically significant vs. control at *p* < 0.01. *** statistically significant vs. control at *p* < 0.001. **** statistically significant vs. control at *p* < 0.0001.

It is expected that 5-FU has cellular activity since it is widely used to treat HT-29 cells, as demonstrated in Figure 3 and Figure 4. The same happened to TAC; although it had better activity only for higher concentrations—50 and 100 µM—it did not show a decrease in cell viability in the remaining concentrations, unlike the first drug. Finally, ETA showed the opposite behavior; besides not having any cellular effect, it possibly favored growth in tumor cells. 

In Table 3, even though TAC also had activity, only 5-FU obtained a value of IC_50_ of 6.10 µM. Duarte et al. [57] studied the activity of 5-FU in the same HT-29 cells and obtained an IC_50_ of 3.79 µM, close to that obtained in this work.

#### 3.3.2. Toxicity Study of CNTs

Before combining the drugs with the samples, it is important to ensure that CNTs are not toxic, so as not to influence future results and to ensure safe conditions for normal cells. As with other nanomaterials, the CNTs can be toxic depending on the synthesis/textural modification, shape, size, and cell type [10,58,59,60,61], so it is necessary to evaluate them individually in the cells under study. Initially, an intermediate study was performed on a few samples with different concentrations, from 2.5 to 80 µg∙cm^−3^, to select the best concentration, which was 40 µg∙cm^−3^.

**Figure 5:**
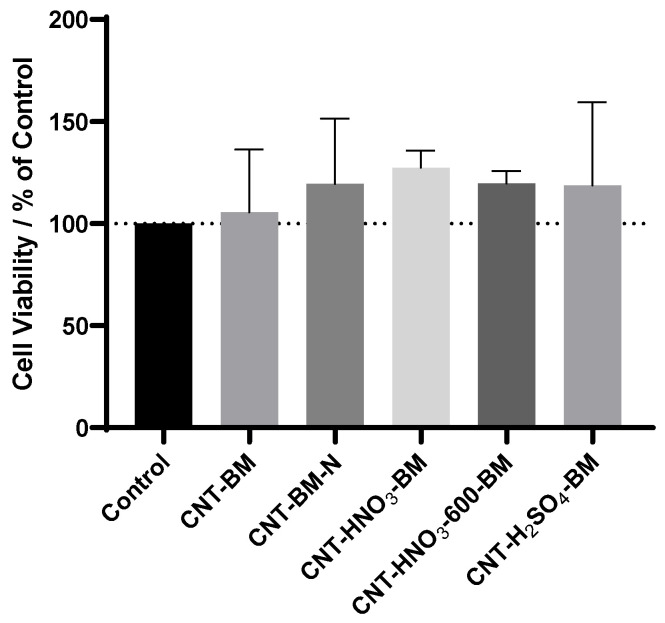
The effects of different samples of CNTs on HT-29 cell viability. Cells were cultured in the presence of increasing concentrations of each drug, and after 48 h, the MTT assay was performed to measure the cellular viability. Values are expressed in percentage of control and represent means ± SEM. Each experiment was conducted three times independently (*n* = 3).

The dispersing agent is not cytotoxic, according to Figure 5. However, it can cause some coloration, without a specific reason, influencing the absorbance values. Therefore, care was taken to present results under the same conditions. Figure 5 compares all the samples with the dispersing agent control, and there is no significant difference between them, which means that no sample is cytotoxic. The increased effect on cell viability might be due to the effect of the dispersing agent, as explained above.

#### 3.3.3. Cell Treatment of Drug/Sample Combinations

First, it was decided to combine the drugs with each sample at a 1:1 ratio, with different drug concentrations, to determine the IC_50_ and compare their activity individually.

CNTs without any functionalization were subjected to mechanical treatments (CNT-BM) in the ball mill. The nanotubes were broken at their ends and could adsorb the drugs onto the graphene sheet layers due to their hydrophobic interactions, while functionalized CNTs contain functional groups on the surfaces that interact with the drugs.

**Figure 6:**
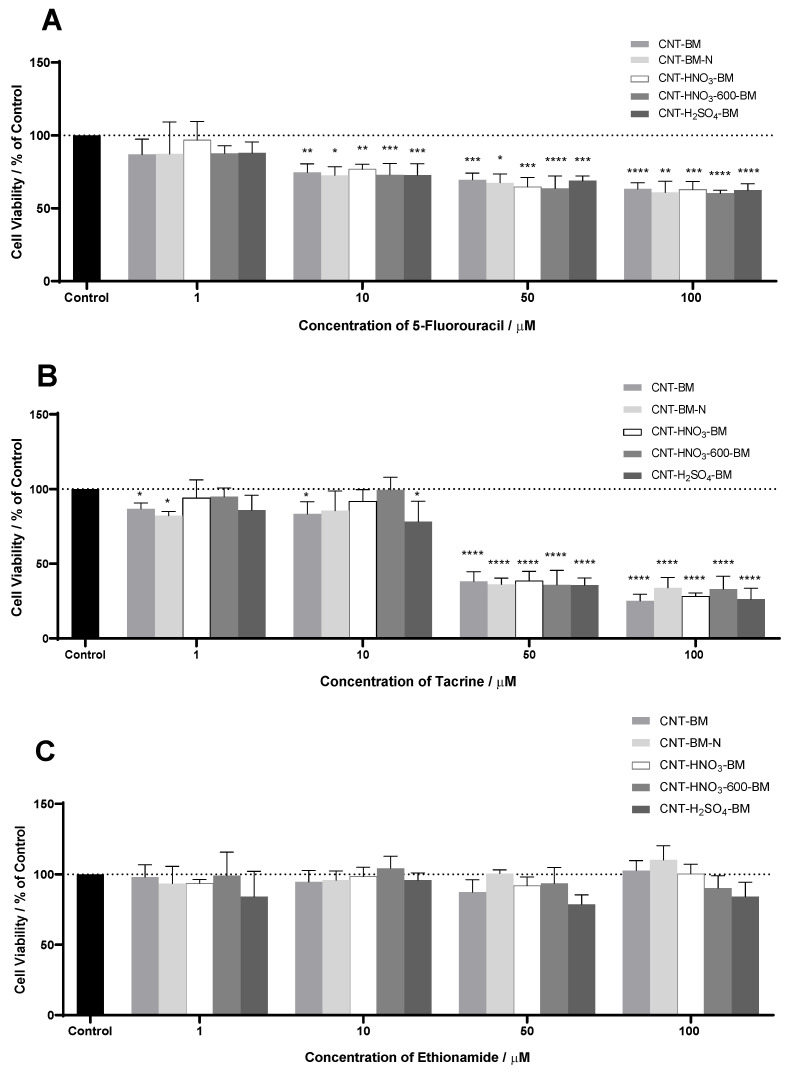
(**A**) The effects of 5-FU on cell viability. (**B**) The effects of TAC on cell viability. (**C**) The effects of ETA on HT-29 cell viability. Cells were cultured in the presence of increasing concentrations of each drug, and after 48 h, the MTT assay was performed to measure the cellular viability. Values are expressed in percentage of control and represent means ± SEM. Each experiment was conducted three times independently (*n* = 3); * statistically significant vs. control at *p* < 0.05. ** statistically significant vs. control at *p* < 0.01. *** statistically significant vs. control at *p* < 0.001. **** statistically significant vs. control at *p* < 0.0001.

5-FU was the only free drug that had an IC_50_ of 6.10 µM. This drug has one aromatic ring with ketones (C=O) and amine groups (N-H), so it is expected to have a more significant interaction (especially involving hydrogen points with the functionalized samples with -COOH, -OH groups, and nitrogen groups) and, consequently, an improvement in the IC_50_. According to Figure 6A, a gradual reduction in cell viability can be seen in all samples with identical behavior as before. In Table 4, the samples with the best activity were CNT-BM-N, CNT-H_2_SO_4_-BM, and CNT-BM, with better IC_50_ values. Contrary to expectations, the oxygenated samples did not significantly interact with 5-FU. Compared these IC_50_ values to free 5-FU—6.10 µM, almost all samples improved their activity, except CNT-HNO_3_-BM, which again denotes an advantage in using nanomaterials. A study by Sharma et al. [62] evaluated the activity of 5-FU encapsulated in polymeric nanoparticles in HT-29 cells and obtained an IC_50_ of 3.7 µM. Another study by Udofot et al. [63] combined 5-FU with different ratios of liposomes, obtaining IC_50_ of 0.41, 6.26, and 8.15 µM. According to these studies, there is an advantage in combining 5-FU with CNTs, which shows a lower IC_50_, as in CNT-BM-N, CNT-HNO_3_-600-BM, CNT-H_2_SO_4_-BM, and CNT-BM samples. 

**Figure 7:**
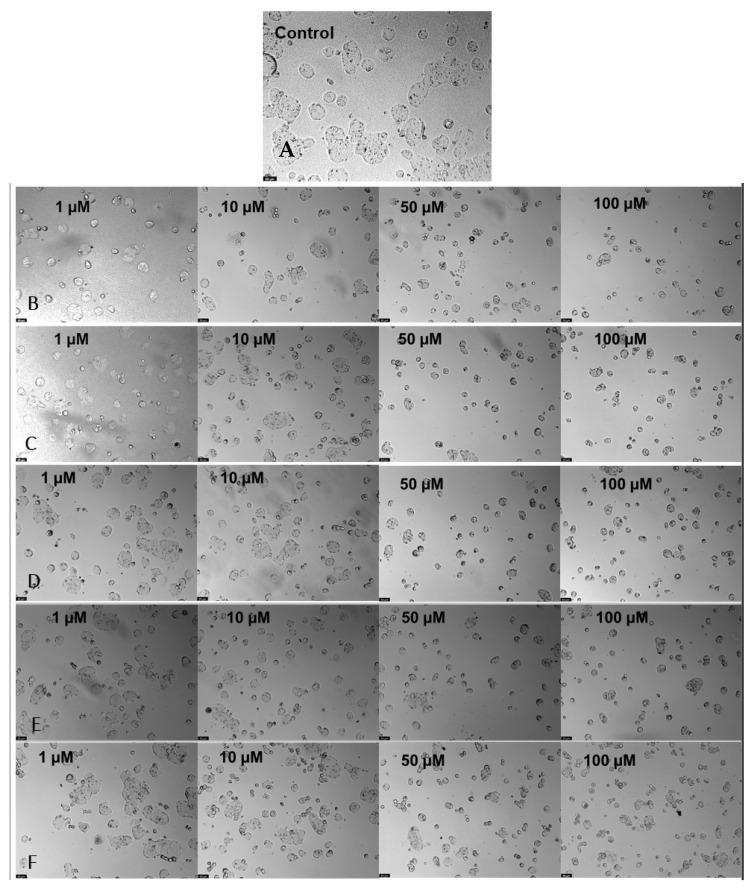
Microscopic cellular visualization of HT-29 cells after 48 h of incubation with a combination of 5-FU at different concentrations of [1–100] µM and the different samples. (**A**) Control. (**B**) CNT-BM. (**C**) CNT-BM-N. (**D**) CNT-HNO_3_-BM. (**E**) CNT-HNO_3_-600-BM. (**F**) CNT-H_2_SO_4_-BM.

Although 5-FU did not bind strongly with the oxygenated samples, an improvement is denoted when combined with CNTs, as shown in Figure 7. These results and the fact that they are better than some other studies make these combinations an alternative application for future work due to the use of low amounts of the drug. Even so, some ways to improve these results are to optimize the drug/sample ratio, which favors its activity, minimizes the IC_50_, and adds other oxygenated groups, providing better binding with 5-FU.

**Figure 8:**
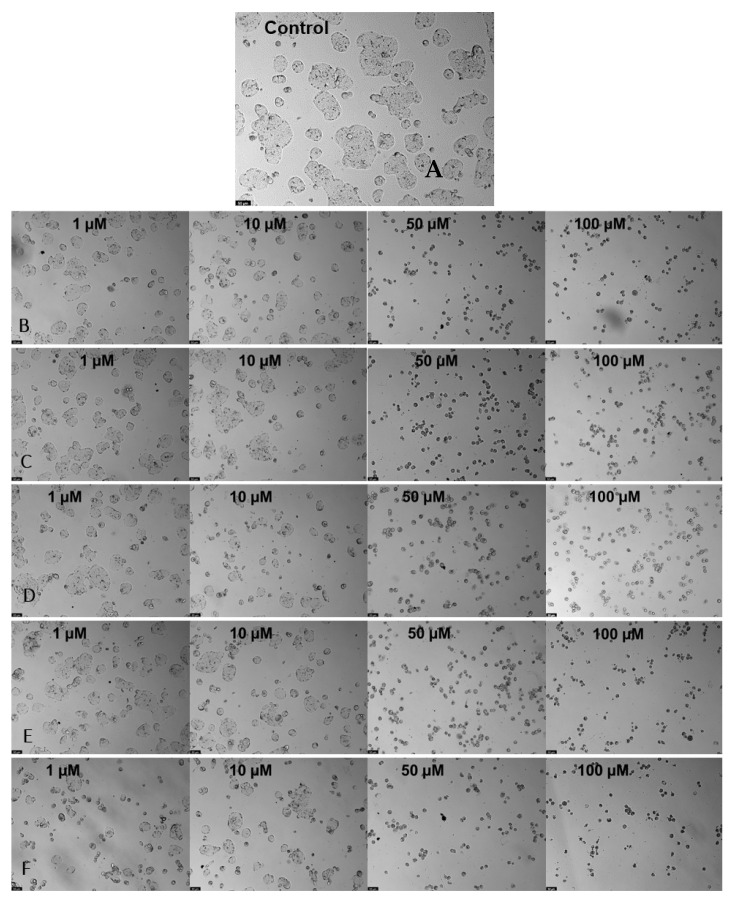
Microscopic cellular visualization of HT-29 cells after 48 h of incubation with a combination of TAC at different concentrations of [1–100] µM and the different samples. (**A**) Control. (**B**) CNT-BM. (**C**) CNT-BM-N. (**D**) CNT-HNO_3_-BM. (**E**) CNT-HNO_3_-600-BM. (**F**) CNT-H_2_SO_4_-BM.

The combination between samples and TAC improved activity in all samples, as shown in Figure 6B and Figure 8, where the concentrations of 50 and 100 µmol∙dm^−3^ reached very significant values in all samples. However, the CNT-BM-N, CNT-H_2_SO_4_-BM, and CNT-BM samples stand out from the lowest concentrations, which, compared with Table 4, denotes much lower IC_50_ values for these samples. This is to be expected since the TAC contains in its molecular form two aromatic rings, one benzene and one amine group, and several C=C bonds, where there is greater interaction with the CNTs via hydrophobic bonds, especially van der Waals forces, that can interact with the different samples. Compared to the activity of TAC alone, this combination indicates a great improvement in general, since it was possible to determine the IC_50_ in this assay. The combinations showed a reduction in cell viability for lower concentrations (1 and 10 µM), unlike previously; even at concentrations of 50 µM, the activity improved in all samples. Since there are no studies combining TAC with other nanomaterials, it is impossible to compare the IC_50_ values, making this work interesting and promising. Still, two possibilities to improve their activity and, consequently, reduce the IC_50_ values to those of 5-FU, are to optimize the drug/sample ratio, which may provide a better binding between both, and try another method of functionalization into CNTs.

**Figure 9:**
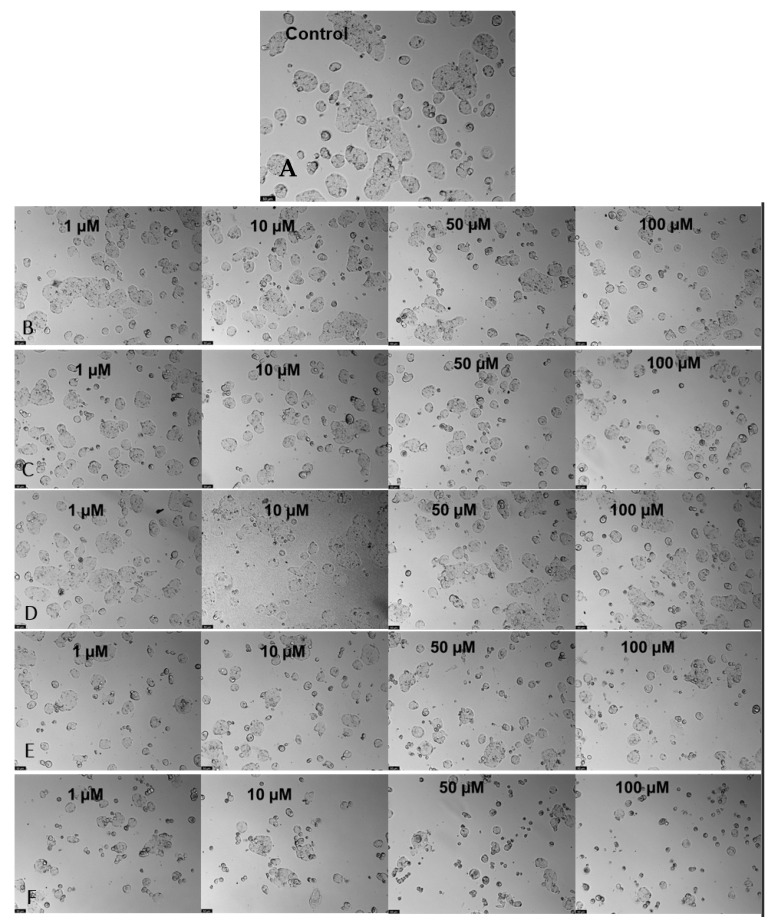
Microscopic cellular visualization of HT-29 cells after 48 h of incubation with a combination of ETA at different concentrations of [1–100] µM and the different samples. (**A**) Control. (**B**) CNT-BM. (**C**) CNT-BM-N. (**D**) CNT-HNO_3_-BM. (**E**) CNT-HNO_3_-600-BM. (**F**) CNT-H_2_SO_4_-BM.

In Figure 6C, some instability in the percentage of cell viability can be seen. The ETA previously showed a gradual increase rather than a decrease in the number of tumor cells. However, in this case, the combination improved its activity; as did the CNT-H_2_SO_4_-BM sample, which stood out the most in these results, as shown in Figure 9. However, this improvement was not enough to determine IC_50_ values. Since ETA is a 1-benzene molecule with one amine group and a few C=C bonds, little interaction with the samples is expected compared to the other drugs. When comparing ETA with TAC, the former has lower hydrophobic forces, which results in less adsorption in the samples. This is in contrast to 5-FU, which despite having lower forces than TAC, can still form stronger forces than hydrophobic forces. Since the combination caused a slight improvement in activity, a possible solution is to optimize the ratio of drug to sample. However, the low activity might be related to the potential weak interaction with the samples, so to better understand its behavior, another solution would be to combine it with other functionalized samples. As mentioned for TAC, no studies have combined other nanomaterials treated in HT-29 cells for this repurposing drug, so comparing the IC_50_ with other studies is impossible.

## 4. Conclusions

The aim of this work was to combine functionalized CNTs with different drugs for application in HT-29 cells. The functionalization procedures with H_2_SO_4_ (CNT-H_2_SO_4_) and melamine (CNT-BM-N) were successful, presenting values of 0.2% S and 4.3% N-groups, respectively. Regarding the amounts of CO and CO_2_, in the CNT sample, 334 and 176 µmol∙g^−1^ were obtained; in the CNT-HNO_3_ sample, 1002 and 440 µmol∙g^−1^ were obtained; and in the CNT-HNO_3_-600 sample, 900 and 360 µmol∙g^−1^ were obtained, confirming the introduction of oxygenated groups on the surface of CNTs. When combined with the CNTs, the reference drug—5-FU—improved its activity, with the samples CNT-BM-N, CNT-H_2_SO_4_-BM, and CNT-BM presenting an IC_50_ of 1.98, 2.50, and 2.87 µM, respectively. TAC, being a repurposing drug, greatly enhanced cell activity in which the samples CNT-BM-N, CNT-H_2_SO_4_-BM, and CNT-BM, obtaining values of IC_50_ of 15.32, 15.81, and 19.73 µM, respectively. On the other hand, ETA had no activity, even combined with the CNTs. The samples that interacted most with the drugs and contributed to improving cell treatment were CNT-BM-N and CNT-H_2_SO_4_-BM. Our work demonstrated that some CNTs combined with 5-FU improved their activity, even compared to other studies in treating HT-29 cells, which indicates the potential for future development of this work. In the repurposing drugs, compared to their activity in free form, only TAC improved its activity when combined with CNTs, which shows how beneficial it is to work with repurposing drugs.

## Figures and Tables

**Table 1:** Textural properties of the prepared samples.

Sample	*d* (µm)	*S_BET_* (m^2^·g^−1^)	*V_pT_*_, P/P0=0.95_ (cm^3^·g^−1^)
CNT	262.8	188	1.49
CNT-HNO_3_	239.1	193	1.68
CNT-HNO_3_-600	261.1	192	1.49
CNT-H_2_SO_4_	261.8	173	1.65
CNT-BM	98.9	275	0.81
CNT-BM-N	123.7	186	0.93
CNT-HNO_3_-BM	50.4	267	0.77
CNT-HNO_3_-600-BM	36.1	281	0.73
CNT-H_2_SO_4_-BM	57.3	246	0.82

**Table 2:** Chemical properties determined by EA, TPD, and TGA.

Sample	EA (wt.%)			TPD (µmol·g^−1^)		TGA (wt.%)	
	N	S	O	CO	CO_2_	VCs	Ash
CNT	0	0	0.2	334	176	4.1	10
CNT-HNO_3_	0	0	1.2	1002	440	9.2	3.0
CNT-HNO_3_-600	0	0	0.5	900	360	4.9	3.5
CNT-H_2_SO_4_	0	0.2	1.4	n.d.	n.d.	7.9	0.9
CNT-BM-N	4.3	0	1.2	n.d.	n.d.	12.9	1.9

n.d.—not determined.

**Table 3:** Cytotoxicity of 5-FU, TAC, and ETA in HT-29 colon cancer cells, in which DMSO was used as a solvent. IC_50_ values are given as the mean.

Sample	5-FU (µM)	TAC (µM)	ETA (µM)
No sample	6.10	>100	>100

**Table 4:** Cytotoxicity of combining each sample with each drug (5-FU, TAC, and ETA) in HT-29 colon cancer cells. IC_50_ values are given as the mean.

Sample	5-FU (µM)	TAC (µM)	ETA (µM)
CNT-BM	2.87	19.73	>100
CNT-BM-N	1.98	15.32	>100
CNT-HNO_3_-BM	6.74	23.31	>100
CNT-HNO_3_-600-BM	3.13	25.72	>100
CNT-H_2_SO_4_-BM	2.50	15.81	>100

## Data Availability

Not applicable.
